# Clinical differences between men and women in a Swedish treatment-seeking population with gambling disorder

**DOI:** 10.3389/fpsyt.2022.1054236

**Published:** 2023-01-04

**Authors:** Louise Miller, Mikael Mide, Elin Arvidson, Anna Söderpalm Gordh

**Affiliations:** ^1^Addiction Biology Unit, Department of Psychiatry and Neurochemistry, Institute of Neuroscience and Physiology, The Sahlgrenska Academy at University of Gothenburg, Gothenburg, Sweden; ^2^Department of Addiction and Dependency, Sahlgrenska University Hospital, Gothenburg, Sweden

**Keywords:** comorbidity, treatment-seekers, pathways, gambling disorder, gender

## Abstract

**Introduction:**

The purpose of this study was to explore clinical differences in Swedish treatment-seeking men and women with gambling disorder (GD). As the prevalence of GD is increasing among women, even though men are still highly overrepresented, the characteristic differences between men and women seeking treatment become increasingly important.

**Method:**

A sample of 204 patients with GD (26.5% women and 73.5% men) at an outpatient clinic were diagnosed using the SCI-GD, screened for comorbid diagnoses using the MINI, and further completed a range of self-report questionnaires measuring demographics, GD, alcohol and other drug problems, symptoms of depression and anxiety, and pathways into gambling problems.

**Results:**

Several characteristics differed between treatment-seeking men and women in our sample. Examples of differences between genders included age, onset age, living situation, duration, alcohol and drug problems, comorbidity, and pathways leading to gambling problems.

**Discussion:**

The most evident difference was that women, in addition to GD, showed more symptoms of anxiety and depression than men, while men had a higher degree of substance use problems compared to women. The differences in clinical features between men and women are important to consider in treatment planning and possibly for future gender-based interventions.

## Introduction

The prevalence of gambling disorder (GD) has globally changed from a range of 0.5–7.6% to 0.3–10.9% in the last 10 years, with an overrepresentation of men (1, 2). In Sweden, prevalence continues to increase. The Swedish Health Authorities report that approximately 0.6% of the population suffers from gambling disorder (3). Gambling disorder is also associated with a high lifetime prevalence of having a comorbid psychiatric disorder, typically depression and substance use disorders (4, 5). In a Swedish nationwide register-based study, it was found that 73% of all patients diagnosed with GD had a co-occurring psychiatric diagnosis and that mortality due to suicide increased 15-fold (6).

Today about 20% of individuals with GD seek treatment for their gambling problems within healthcare services (7). This is an increase from the last report where 9.9% of patients with GD seek treatment (8, 9) and women present a lower chance of being in treatment (10). Considering this, the different clinical characteristics in men and women seeking treatment are still unknown, and knowledge needs to be updated. Even though women have increased their gambling habits and also show a increased prevalence of GD within the last decade (11, 12) men still gamble more frequently, for more money and they show a higher prevalence of gambling related problems than women do (13). In Sweden, it has been estimated that 0.2% of women and 0.8% of men have a severe gambling disorder and that more women than men proportionally stand for new cases i.e., number of individuals progressing from no problems or low risk to gambling problems or gambling disorder (3, 11).

Studies specifically comparing treatment-seeking men with women are limited [for review, refer to Gartner et al. (1)]. When only taking such studies into account, considerably more men than women seek treatment for their gambling disorder (14–19). The largest differences were observed in treatment-seekers from Britain, where 92.5% were men and 7.5% were women (20). This is comparable to a study in a treatment-seeking population in Sweden in where 80% were men and 20% were women (19).

Moreover, it has been reported that woman are older than men when they enter treatment (15, 17, 18, 20–22), that they tend to progress to gambling disorder faster and that they seek treatment earlier than men (14, 15, 21–29). However, in a sample of 2,256 gamblers seeking treatment, gender contribution to problem progression did not differ when age at onset and age of gambling initiation were taken into account (30). Treatment-seeking women also have more comorbidity than men. Women report a higher prevalence of both affective and anxiety disorders (18–21, 24, 26, 29, 31–34) and have more general psychopathology (16). In contrast, men report more alcohol and other drug problems compared to women (14, 18–21, 32, 33, 35, 36). Interestingly, women tend to experience comorbid disorders before the onset of gambling, while men tend to experience other disorders after the first onset of problem gambling (28, 37).

Most studies also report that women often live alone, i.e., are likely to be divorced or widowed (21). They report that feelings of loneliness can trigger gambling initiation (35); and they are more likely to be retired, unemployed, or outside the workforce (20, 22, 36) or have problems with their professional life (34). However, the opposite has also been observed, with treatment-seeking women more likely to be married, living with family, and having dependent children (17).

Furthermore, treatment-seeking women engage more in casino gambling and bingo than men (18, 21, 29, 38) and are more likely to use electronic gaming machines (17, 26), with a preference for non-strategic forms of gambling (22, 24). Treatment-seeking men more often engage in sports betting (19–21). In treatment-seeking individuals, it has also been reported that female gamblers often report being victims of family violence (21, 34, 39) and that they, to a larger extent than men, had been exposed to childhood maltreatment (40).

Men and women also seem to differ in their pathways into GD. Using the Blaszczynski model (41), which presents three potential pathways into a GD, it has been suggested that women more likely than men start to gamble through the “emotionally vulnerable” pathway, i.e., they start to gamble primarily to escape aversive mood states. More so than women, men start to gamble through the “antisocial impulsivists” pathway, i.e., because of factors such as heightened impulsivity, antisocial personality traits, and comorbid substance use (42, 43). No gender differences have been found for the third pathway, the “behaviorally conditioned,” which is described as a pathway with the absence of psychopathology, where it is theorized that gambling is initiated for recreation or socialization reasons.

Based on the increasing prevalence of GD in women (11, 12), even though men are still overrepresented, and more women than men proportionally stand for new cases of problem gamblers in Sweden (11, 44), we aim to explore sociodemographic characteristics, clinical correlates, co-morbidity, and the pathways into gambling problems (according to the Blaszczynski and Nower pathway model). We will study a sample of men and women seeking treatment at Sweden’s largest outpatient clinic for gambling disorders.

## Materials and methods

### Study design

Our data were collected from individuals seeking treatment at the Clinic for Gambling Addiction and Screen Health between the first day of the opening of the clinic in May 2018 and May 2022. The demography, gambling severity, and prevalence of other psychiatric diagnoses were assessed. We also mapped other clinically relevant outcomes such as additional addictive behaviors, quality of life, and gambling-related cognitive distortions among these individuals. The information was obtained from several semi-structured interviews and standardized questionnaires.

### Participants

The participants (*n* = 208) were recruited from the Clinic for Gambling Addiction and Screen Health at Sahlgrenska University Hospital in Gothenburg, Sweden, the largest public health outpatient facility offering treatment for pathological gambling in Region Västra Götaland in Sweden. Region Västra Götaland has 1.6 million inhabitants, with the clinic located in Gothenburg with its population of approximately 1 million. The clinic welcomes both patients with GD and gaming disorder from 16 years of age, and the treatment is based on cognitive behavioral therapy. Patients were either self-referred or referred by a physician or other healthcare professional to the clinic. No specific inclusion criteria were set. All patients that attended their first assessment at the clinic were asked to participate in the study. Patients were excluded if they did not fulfill the criteria for GD.

### Procedure

On the first visit to the clinic, all patients were assessed with a semi-structured anamnestic interview and a semi-structured diagnostic interview for diagnosing GD. Additionally, sociodemographic data and several questionnaires were given to measure, e.g., various aspects of mental health and quality of life. Patients were also assessed with a psychiatric structured diagnostic interview. This was only done for patients that did not have another psychiatric contact outside the clinic. On their first visit to the clinic, the patients were informed about the study and approved participation by signing an informed consent form in connection with their visit. The study was approved by the Swedish Ethical Review Authority, dnr 764-18, and was conducted according to the 1964 declaration of Helsinki.

### Measures

#### Clinical interviews

*Structured Clinical Interview for Gambling Disorder (SCI-GD)* is a semi-structured guide for interviewing patients with suspected GD. It is based on the diagnostic criteria for GD in the latest version of the DSM-5 (45). If four or more of the criteria were met then the patient was diagnosed with GD. Fulfilling four to five criteria counts as mild GD, 6–7 as moderate GD, and 8–9 as severe GD (46).

*The Mini-International Neuropsychiatric Interview (M.I.N.I*.) is a brief structured interview based on diagnostic criteria in DSM-5 and ICD-10. The therapist asks specific questions and the patient answers all questions with “yes” or “no.” The instrument is validated against other psychiatric measurements with good results (47). The M.I.N.I. has also been studied in a Swedish context (48) and is recommended by Swedish health authorities for use in addictive care (49).

*Anamnestic interviews* assess tobacco use, drug use, and other psychiatric diagnoses besides gambling. Drug use and psychiatric diagnoses were assessed using the MINI-psychiatric interview (47, 48), AUDIT (50), and DUDIT (51) questionnaires. Information related to gambling was also collected; age of gambling onset, how many years since gambling became a problem, the function of gambling (e.g., economic reasons), and the dominating type of gambling (e.g., sports betting).

#### Self-report questionnaires

*The NORC Diagnostic Screen for Gambling Problems (NODS latest 30 days*) is a self-report questionnaire measuring the severity of the gambling problem based on the diagnostic criteria in DSM-5. The instrument consists of 17 questions with response alternatives “yes” or “no.” The severity of gambling problems is classified into three categories based on the number of questions answered “yes”: risk gambling (1–2 yes), problem gambling (3–4 yes), and pathological gambling (5–10 yes) (52). Three out of 17 questions do not give any points, and one yes in three specific groups of questions (e.g., questions 14, 15, or 16) gives one point. This explains the inclusion of 17 questions but only 10 points (“yes”) maximum. It is considered to have good internal validity and clinical applicability (53).

*The Patient Health Questionnaire (PHQ-9)* consists of nine items screening for symptoms of depression during the last 2 weeks. PHQ-9 is developed according to diagnostic criteria in DSM-IV, and the total score can be used to assess the severity of depressive symptoms. Based on the total score, the level of severity is classified as none (0–4), mild (5–9), moderate (10–14), moderately severe (15–19), or severe (20–27). PHQ-9 has good validity for the detection of the severity of depression (54).

*Generalized Anxiety Disorder Assessment (GAD-7*) was developed as an instrument to measure the presence and grade of the symptoms of anxiety. It is a seven-item questionnaire screening for symptoms during the last 2 weeks. The total score is 21, with cut-off points at 5, 10, and 15, indicating minimal (0–4), mild (5–9), moderate (10–14), and severe (15–21) levels of anxiety (55).

*Gambler’s beliefs questionnaire (GBQ)* is a measure of cognitive distortions in individuals with problematic gambling. The questionnaire consists of 20 gambling-related statements regarding thoughts connected to gambling. Higher scores indicate more irrational cognitions related to gambling (56). The GBQ has demonstrated reliability through its excellent internal consistency (56–58).

*Alcohol Use Disorders Identification Test (AUDIT)* screens for alcohol-related problems and identifies individuals with harmful use of alcohol. It consists of 10 items, divided into three areas: alcohol consumption, symptoms of dependence, and negative consequences of alcohol consumption. The maximum score is 40, with a cut-off score of 6 for women and 8 for men, indicating hazardous or harmful drinking (50).

*Drug Use Disorders Identification Test (DUDIT)* screens for use of illicit drugs and events of drug-related consequences. Similar to AUDIT, it is a 10-item instrument with a maximum score of 40. The questions are categorized into three areas: drug use, dependence symptoms, and negative consequences of drug use. DUDIT scores of one or more for women and three or more for men indicate problematic drug use (51).

*Brunnsviken Brief Quality of life scale (BBQ)* measures an individual’s subjective quality of life in a clinical setting. It is divided into six different life areas such as “view on life,” “creativity,” and “friends and friendship.” A total score is 96 with higher scores indicating a higher perceived quality of life, and a score of 52 or lower is considered a cut-off indicating poorer quality of life (59).

*Difficulties in Emotion Regulation Scale (DERS-16)* measures difficulties in the regulation of emotions. It consists of 16 statements concerning reactions to emotional discomfort. Response alternatives are graded from 1 (almost never) to 5 (almost always). The scoring ranges from 16 to 80, with higher scores representing larger difficulties in emotion regulation. DERS-16 is divided into five subscales: clarity, goals, impulse, strategies, and non-acceptance (60).

*The Gambling Pathways Questionnaire (GPQ)* proposes three pathways for identifying etiological subtypes of problem gamblers. It consists of 48 statements and the response alternatives are graded from 1–6 points (strongly disagree 1 point to strongly agree 6 points).

The 48 responses allocate to one of three sub-scales which defines a specific pathway. The pathways are described as “behaviorall conditioned gamblers,” “emotionally vulnerable gamblers,” and “antisocial/impulsivist gamblers” (61).

*The World Health Organization adult ADHD self-report scale (ASRS-V1.1 screener)* is a screening tool, identifying adult individuals with symptoms of ADHD. Part A contains six items that are the most predictive symptoms of ADHD and are based on symptoms that are described in the DSM-IV with a 5-point response scale ranging from “never” to “very often” (62). Four or more responses at a specific severity level are considered indicative of a possible ADHD diagnosis. Part B contains 12 additional questions, which were not used here.

*The demographic data questionnaire* acquires several demographic aspects from the participants including age, sex, educational level, civil status, living situation, and current occupation.

### Missing data

Informed consent to participate in the study was received from a total of 208 patients during the study period. Of the 208 participants, one had a missing result on the SCI-GD and was excluded as it was not possible to confirm a diagnosis of GD. Similarly, another three were excluded as their SCI-GD score was < 4 and thus did not fulfill the criteria for GD. This left a total of *n* = 204 participants for analysis. Due to clinical considerations, the battery of measurements was changed during the course of the study. As such, the GPQ (*n* = 93) and ASRS (*n* = 86) were not administered to all participants. The MINI (*n* = 161) was also not administered to all participants due to clinical considerations. Frequencies of unanswered questionnaires for all other measures were between 5 and 11%.

### Data analysis

All analyses were performed using IBM SPSS Statistics version 28. To test the normality of continuous variables, a series of Shapiro–Wilk tests (63, 64) were performed. These indicated non-normality for all variables except the GBQ. Further analysis of histograms showed that AUDIT, DUDIT, duration of problems, years from onset to problems, and NODS were severely skewed and, thus, violated assumptions for parametric statistics. All other variables were close to normally distributed and deemed to not violate assumptions. Participants could give multiple answers regarding which gambling types they engaged in, and their reasons for gambling. These answers were clustered into five categories. For gambling types: online slots/casino, sports betting (online and offline), gambling in a physical store (bingo, slots, poker, horses), day trading, and others. For gambling reasons: financial, escape, excitement, habit, and self-harm. Frequencies for each gambling type and reason were reported in percentages. A Pearson Chi-square test was used to test possible differences between genders for categorical demographic variables and categorical gambling behaviors except in cases where > 20% of cells had an expected count less than five (reason: self-harm, gambling type: physical store, gambling type: day trading, occupation), where Fisher’s exact test was used instead (65). Possible gender differences in the level of alcohol and other drug problems were evaluated by comparing frequencies of categorized problem levels using Chi-square tests and also by comparing raw scores on AUDIT and DUDIT using Mann–Whitney tests (66). The Mann–Whitney test was also used to test for differences between the duration of problems, years from onset to problems, and the NODS. To examine possible gender differences regarding age and age of gambling onset, an independent *t*-test was used. When assessing whether there were any gender differences between different clinical measures (PHQ-9, GAD-7, DERS-16, GBQ, and BBQ), age and possible ADHD were decided to be possible confounders based on a review of the literature (67–71). To assess possible differences and also control for confounders, Analysis of Covariance (ANCOVA) was used. Participants were coded as having possible ADHD if they either had screened positively for ADHD on ASRS or the MINI or had an ADHD diagnosis in their medical records. No correction was made due to multiple testing, as the study is of exploratory nature and does not include any confirmative hypotheses.

## Results

### Sociodemographic characteristics

Sociodemographic characteristics of the total participants, as well as for women and men separately, together with test statistics, *p*-values, effect sizes, and odds ratios are reported in Table 1. Notably, the sample consisted of fewer women (*n* = 54) than men (*n* = 150). The women were older than the men, were more often single parents, and less often lived together with a partner without children. There was no difference between genders regarding the prevalence of possible ADHD.

**TABLE 1:** Demographics of total sample, women, and men.

	Total sample (*n* = 204)	Women (*n* = 54)	Men (*n* = 150)	Test-statistic^5^	*p*-value	Effect size (odds ratio/Cohen’s *d*)^6^
Age *M* (*SD*)	36.1 (10.2)	40.5 (10.0)	34.5 (9.8)	*t* = 3.9	< 0.001*	*d* = 0.61
Education %				X^2^ = 4.4	=0.22	–
Less than high school	18.6	17.6	18.9			
High school	57.7	51.0	60.1			
Occupational training	3.6	7.8	2.1			
University	20.1	23.5	18.9			
Occupational status %				–	=0.335	–
Working	70.8	69.8	71.1			
Sick-leave	14.4	13.2	14.8			
Unemployed	6.9	3.8	8.1			
Other	7.9	13.2	6.0			
Living situation %				X^2^ = 22.1	< 0.001*	
Alone	28.4	28.3	28.4			OR = 1.0
With partner	20.4	3.8	26.4			OR = 0.1
With relatives/friends	11.9	13.2	11.5			OR = 1.2
Single parent	10.9	24.5	6.1			OR = 5.0
With partner and children	26.9	28.3	26.4			OR = 1.1
Other	1.5	1.9	1.4			OR = 1.4
Possible ADHD %				X^2^ = 0.1	= 0.702	–
Yes	24.0	25.9	23.3			
No	76.0	74.1	76.7			
Nicotine use %				X^2^ = 0.4	=0.524	–
Yes	65.1	68.6	63.9			
No	34.9	31.4	36.1			
AUDIT *M* (*SD*)^1^	6.2 (6.2)	4.0 (4.4)	6.9 (6.6)	*U* = 4617.0	< 0.001*	*d* = 0.47
Harmful alcohol use %^2^				X^2^ = 0.02	= 0.902	–
Yes	29.3	30.0	29.1			
No	70.7	70.0	70.9			
DUDIT *M* (*SD*)^3^	2.7 (7.5)	0.9 (3.3)	3.3 (8.4)	*U* = 3915.0	= 0.042*	*d* = 0.32
Problematic drug use %^4^				X^2^ = 4.3	= 0.038*	OR = 0.02
Yes	17.6	8.0	21.0			
No	82.4	92.0	79.0			

Data is presented as means and standard deviations *M* (*SD*) and in percent (%).

^1^Alcohol use disorders identification test.

^2^AUDIT scores ≥ 6 for women and ≥ 8 for men indicate potential harmful alcohol consumption.

^3^Drug use disorders identification test.

^4^DUDIT scores ≥ 1 for women and ≥ 3 for men indicate problematic drug use.

^5^Test statistics are either X^2^, *t*, or *U*, depending on statistical test. For Fisher’s exact test there is no test statistic.

^6^Effect size is reported as odds ratios for categorical variables and Cohen’s *d* for continuous variables. Odds ratios are reported for women to men. Effect size is only reported for significant effects. *Statistically significant.

The majority of the participants used nicotine in some form and harmful alcohol based on the AUDIT scale cut-offs was also common. There was no difference between genders in the frequency of harmful use, but when comparing raw scores on the AUDIT men had a higher score than women. Regarding problematic use of other drugs, men more often had a problematic use and scored higher on the DUDIT.

### Gambling behaviors

Gambling behaviors of the total participants, as well as women and men separately, together with test statistics, *p*-values, effect sizes, and odds ratios are reported in Table 2. Men and women had the same degree of gambling problems according to the SCI-GD and the NODS. Among all participants, a severe form of GD was the most common. The most common dominant gambling type for both men and women was an online casino. The opposite was true for online sports betting, which none of the women stated as their dominant gambling type. It was also more common for men to fall into the “other” category.

**TABLE 2:** Gambling behaviors and pathways for total sample, women, and men.

	Total sample (*n* = 204)	Women (*n* = 54)	Men (*n* = 150)	Test-statistic^4^	*p*-value	Effect size (odds ratio/Cohen’s *d*)^5^
Dominant gambling type^1^ %						
Online slots/casino	66.2	94.4	56.0	X^2^ = 26.2	< 0.001*	OR = 13.4
Online sports betting	24.0	–	32.7	X^2^ = 23.2	< 0.001*	–^6^
Physical store	6.9	3.7	8.0	–	= 0.363	–
Day trading	4.9	–	6.7	–	= 0.066	–
Other	9.3	1.9	12.0	X^2^ = 4.8	= 0.028*	OR = 0.1
Reasons for gambling^1^ %						
Economic	63.2	57.4	65.3	X^2^ = 1.1	= 0.300	–
Escape	58.8	63.0	57.3	X^2^ = 0.5	= 0.471	–
Excitement	38.7	27.8	42.7	*X*^2^ = 3.7	= 0.054	–
Habit	10.8	9.3	11.3	*X*^2^ = 0.2	= 0.674	–
Self-harm	4.4	7.4	3.3	–	= 0.249	–
Age of gambling onset *M* (*SD*)	21.0 (10.4)	27.2 (12.9)	18.8 (8.2)	*t* = 4.5	< 0.001*	*d* = 0.87
Duration of problem *M* (*SD*)	7.9 (7.5)	6.5 (7.1)	8.4 (7.5)	*U* = 4803.5	= 0.028*	*d* = 0.26
Years onset to problem *M* (*SD*)	7.3 (8.1)	6.9 (9.9)	7.4 (7.3)	*U* = 4739.5	= 0.028*	*d* = 0.07
NODS-30^2^ *M* (*SD*)	6.0 (3.0)	6.1 (3.1)	6.0 (2.9)	*U* = 3418.0	= 0.551	–
Gambling disorder severity %				X^2^ = 0.1	= 0.936	–
Mild	13.7	13.0	14.0			
Moderate	38.7	40.7	38.0			
Severe	47.5	46.3	48.0			
Gambling pathway %^3^	(*n* = 93)	(*n* = 29)	(*n* = 64)	X^2^ = 11.1	= 0.004*	
Behavioral conditioned	50.5	44.8	53.1			OR = 0.7
Emotional vulnerable	16.1	34.5	7.8			OR = 6.2
Antisocial/impulsivist	33.3	20.7	39.1			OR = 0.6

Data is presented as means and standard deviations *M* (*SD*) and in percent (%).

^1^Total percentages exceed 100% for dominant gambling type and reasons for gambling as participants could give several answers.

^2^The NORC diagnostic screen for gambling problems (NODS) (latest 30 days).

^3^As the gambling pathways questionnaire was only administered to a limited number of the participants the number of participants were lower for this analysis.

^4^Test statistics are either X^2^, *t*, or *U*, depending on the statistical test. For Fisher’s exact test there is no test statistic.

^5^Effect size is reported as odds ratios for categorical variables and Cohen’s *d* for continuous variables. Odds ratios are reported for women to men. Effect size is only reported for significant effects.

^6^Odds ratio could not be calculated as the number of women stating sports betting were zero.

*Statistically significant.

The most stated reason for gambling in all participants was economic reasons, followed by escape, excitement, habit, and self-harm. Women and men did not differ on any of the reasons for gambling, although there was a trend approaching significance regarding excitement. Women had a later gambling onset, a shorter duration of gambling problems, and fewer years between their gambling onset and the time when gambling became a problem.

Pathways to gambling were measured by the GPQ in a smaller subset of the sample, women (*n* = 29) and men (*n* = 64). There was a significant difference between gambling pathways between genders. The most common type for both genders was “behaviorally conditioned gamblers.” The most notable difference was that women were much more likely to be categorized as “emotionally vulnerable gamblers” than men. Men were also slightly more likely to be categorized as “antisocial/impulsivist gamblers” than women.

### Clinical measures

Results from the ANCOVAs assessing the differences in clinical measures between women and men, together with unadjusted and adjusted means, *F*-values, *p*-values, and effect sizes are reported in Table 3. The level of depressive symptoms in the total sample, as measured by the PHQ-9, was *M* = 13.8 (*SD* = 7.0), which can be considered a moderate level. Women had more depressive symptoms than men. Covariates included in this analysis were age (*F* = 0.0, *p* = 0.963) and ADHD (*F* = 16.8, *p* < 0.001).

**TABLE 3:** ANCOVA results between women and men for depression (PHQ-9), anxiety (GAD-7), emotional instability (DERS), gambling related cognitive distortions, (GBQ-SE), and quality of life (BBQ): *p*-values, means, and adjusted means with 95% confidence intervals.

Measure		Women	Men	*F*-value	*p*-value	Effect^6^ size
	Unadjusted	15.6	13.2			
	95% CI	(13.8–17.4)	(12.014.3)			
PHQ-9^1^				4.7	= 0.036*	d = 0.36
	Adjusted	15.6	13.2			
	95% CI	(13.7–17.5)	(12.1–14.3)			
	Unadjusted	12.2	10.3			
	95% CI	(10.5–14.0)	(9.4–11.3)			
GAD-7^2^				4.3	= 0.039*	d = 0.33
	Adjusted	12.3	10.3			
	95% CI	(10.7–13.9)	(9.4–11.3)			
	Unadjusted	45.6	42.4			
	95% CI	(40.8–50.3)	(39.5–45.3)			
DERS^3^				2.0	= 0.155	–
	Adjusted	46.2	42.1			
	95% CI	(41.4–51.0)	(39.3–45.0)			
	Unadjusted	71.8	71.8			
	95% CI	(66.1–77.7)	(68.0–75.6)			
GBQ^4^				0.0	= 0.958	–
	Adjusted	72.0	71.8			
	95% CI	(65.7–78.3)	(68.1–75.5)			
	Unadjusted	34.9	38.0			
	95% CI	(28.8–41.0)	(34.4–41.5)			
BBQ^5^				0.7	= 0.414	–
	Adjusted	34.9	37.9			
	95% CI	(28.8–41.1)	(34.3–41.5)			

^1^Patient health questionnaire.

^2^Generalised anxiety disorder assessment.

^3^Difficulties in emotion regulation scale.

^4^Gamblers’ beliefs questionnaire.

^5^Brunnsviken brief quality of life scale.

^6^Cohen’s *d*, calculated with differences between adjusted means and raw score standard deviations.

*Statistically significant.

Regarding anxiety symptoms, as measured by the GAD-7, the total sample mean was *M* = 10.8 (*SD* = 6.0) which is also a moderate symptom level. As revealed by an ANCOVA, the women also had more anxiety symptoms than the men. Covariates included in this analysis were age (*F* = 0.0, *p* = 0.858) and ADHD (*F* = 20.4, *p* < 0.001).

The mean value for emotional dysregulation, as measured by the DERS-16, in the total sample, was *M* = 43.2 (*SD* = 17.0). There was no difference between men and women. Covariates included in this analysis were age (*F* = 1.1, *p* = 0.299) and ADHD (*F* = 15.3, *p* < 0.001).

There was also no difference between genders regarding the level of cognitive distortions related to gambling, as measured by the GBQ. Covariates included in this analysis were age (*F* = 0.0, *p* = 0.961) and ADHD (*F* = 5.8, *p* = 0.017). The mean for the total sample was *M* = 71.8 (*SD* = 21.9).

Finally, the total sample on average showed a clinical level of life dissatisfaction according to the BBQ, *M* = 37.2 (*SD* = 21.2). Frequencies of participants with a clinical level of life dissatisfaction (below the cut-off score) were 81.0% for the total group, 85.4% for women, and 79.4% for men. There was no difference between men and women on mean BBQ scores. Covariates included in this analysis was age (*F* = 0.0, *p* = 0.938) and ADHD (*F* = 4.3, *p* = 0.039).

## Discussion

The present study analyzed sociodemographic characteristics, comorbidity, and pathways in 204 treatment-seeking men and women with GD. On one hand, men seeking treatment at our clinic were around 5 years younger than the women; they started gambling about 10 years earlier and also had problematic gambling behavior for about 2 years longer. Women, on the other hand, developed a gambling problem faster than men even though they had gambled for a shorter duration and had a gambling problem for less time. This pattern has been seen in previous studies of clinical populations. The Ronzitti et al. (20) study found very similar results demonstrating that treatment-seeking men were about 6 years younger than women, they started gambling around 8 years earlier and had a problematic gambling behavior for about 3 years longer than the women. Similar age characteristics were also seen in the studies cited in the introduction (14, 15, 17, 18, 21, 22) finding altogether an average that the men were 9 years younger, started gambling 10 years earlier, and had gambling problems 6 years longer than the women. The men in our study started to gamble 10 years earlier than the women. This needs to be taken into consideration when looking at the pathway into GD. Early initiation of gambling behavior, in men specifically, is a well-known factor for GD, and specific awareness for prevention efforts is warranted.

The faster progression of women into a GD than men is called a telescoping effect and is well documented in previous research (18, 25, 30, 72, 73), although men have also been found to be “telescoped” (74). The same telescoping effect has previously also been seen to differ between men and women in several treatment-seeking populations (15, 20–24, 26–29, 38) except for one study in which both ages of onset and gambling initiation was taken into consideration (30). This is also interesting in light of the increase in GD prevalence seen in women (3, 11, 13) and that concomitantly fewer women seek treatment for GD than men (14–19). However, a small percentage increase is noted between a previous study from Sweden in where 20% were treatment-seeking women (19) compared to our finding that 26.5% women sought treatment in 2022. It has yet to be investigated whether this finding is due to the increased prevalence of GD or other factors, such as lessened stigmatization around the disorder.

Furthermore, our study found that more women than men lived without a partner and that women were more likely to be single parents than men. Other studies have found similar results. Echeburúa et al. (21) found in their clinical population that women more often lived alone and were more likely to be divorced or widowed. It has also been reported that loneliness is the main trigger of gambling initiation for women (38, 75). On the contrary, the opposite has been seen in a study from Australia comparing 1,520 problem gamblers seeking treatment where the women were more likely to be married than the men (17). Nevertheless, most studies report no differences in marital status between men and women seeking treatment for GD (14–16, 76). However, the majority of single-parent households are headed by mothers (77), and, in line with this, more treatment-seeking women than men were single parents in our study. In Sweden, it is estimated that 36.2% of the Swedish population lives in single households, with more women than men living alone (78). This hypothetically may indicate that women need a specific treatment plan directed toward single-parent households.

We further found that the dominant style of gambling for women was online slot machines. Gambling has previously been known to be a more social activity for men than for women (17), and this may influence the context in which men and women choose to gamble. Treatment-seeking women choose to gamble online while men, in addition to online gambling, also gamble in person (17). The choice of online gambling for women may be a result of the stigmatization women still experience over their gambling (12).

Albeit lately, both men and women have been found to gamble online (79), treatment-seeking women specifically choose this method (19). This is in line with our results showing that even though the most common dominant gambling type for both men and women is online gambling, online gambling was more common among women (94.4%) than men (56.0%). Since online gambling has increasingly become a greater and more substantial problem, specifically in individuals with gambling problems in the last 2 years, as a consequence of COVID-19 (80) clinicians should assess and pay extra attention to this gambling type in treatment.

We further found that women showed significantly more depressive and anxiety symptoms than men. In a meta-analysis of the treatment-seeking problem and pathological gamblers, depression was one of thirteen predictors of gambling problems (81). However, the meta-analysis did not make any gender-specific associations. When comparing men and women seeking treatment for GD and their different comorbid symptoms women are more affected by depressive symptoms, they are more anxious, and they have poorer self-esteem than men (18–21, 24, 26, 29, 31–34).

Moreover, also in line with previous studies (18–21, 32, 33, 35, 36), the men in our study were found to have more problematic use of both alcohol and other drugs than women. Interestingly, it has been seen that treatment-seeking women tend to experience comorbid disorders, such as the experience of anxiety or depression before the onset of gambling problems (37), while men tend to experience other disorders after the first onset of problem gambling (28). This was, however, not investigated in our study.

It has been established that patients with concurrent depression present with greater severity of GD and have a higher anxiety level and more cognitive impairments (82, 83). It has also been seen that depression predicts GD severity (84) and that individuals who gamble to moderate their mood have a higher incidence of depression than others (85). Nevertheless, we cannot know from our data if comorbidity with depression/anxiety/substance use is a consequence rather than a cause.

Even though we only looked at pathways in a smaller subset of the patients, we saw a gender difference in the Blaszczynski and Nower gambling pathway model (41). First, the majority of both men (53.1%) and women (44.8%) were “behavioral conditioned gamblers.” This is the most common pathway, in which one initiates gambling activities for social or entertainment reasons and develops a problem as a result of behavioral conditioning. Second, 34.5% of the women fall into the pathway of “emotional vulnerable gamblers” compared to only 7.8% of the men. People falling into this pathway are described as having higher levels of comorbidity with anxiety, depression, and substance use/abuse and with the escape of negative mood states as the motive to gamble. Third, 20.7% of the women were categorized as “antisocial/impulsivist gamblers” compared to 39.1% of the men.

This pathway is a subgroup of the “emotional vulnerable gamblers” that also exhibit impairments in neuropsychological functioning, attention deficit disorders, and impulsivity problems, which may be the reason why they show gambling problems [for review, refer to Dowling et al. (81)]. In the revised pathway model, anxiety, depression, childhood maltreatment, impulsivity, risk taking, and antisocial traits presided gambling problems for those categorized as “antisocial/impulsivist gamblers” (42). In a study looking at non-treatment-seeking problem gamblers, it was found that women were significantly overrepresented in both the “behaviorally conditioned gamblers” (36.2%) and “emotional vulnerable gamblers” (62.3%) pathways. No female overrepresentation was found in the “antisocial/impulsivist gamblers” pathway (86). Furthermore, in an earlier study, it was found that among 2,670 problem gamblers participating in a self-excluding program significantly more women were found in the “emotionally vulnerable gamblers” pathway compared to the other two pathways and significantly more men were found in the “antisocial/impulsivist gamblers” pathway (22). These studies all support our findings that more women than men were described as “emotional vulnerable gamblers” and more men were described as “antisocial/impulsivist gamblers.” Assessing the GPQ in treatment planning may identify men and women that benefit from different strategies in treatment, i.e., women with more stress-relieving coping strategies and men with more impulse control strategies.

Besides the Blaszczynski model, other aspects of differences between men and women have been recognized in the literature. Sancho et al. (69) found in a treatment-seeking population that more men than women had emotion regulation deficits such as non-acceptance of emotions and impulse control. These deficits could explain why close to twice as many men than women (39.1 vs. 20.7%) in our patient sample fell into the category “antisocial/impulsivist gamblers” from the Blaszczynski model, although emotion regulation was not studied here.

This study has several strengths but also limitations. The first strength is that this study deepens the understanding and supports previous literature on gender differences in a clinical population. Furthermore, our patient group represents a true treatment-seeking population in specialized outpatient care. All patients were recruited to the study when they attended their first visit to the clinic after either self-referral or referral by a physician or other healthcare professional. All patients were diagnosed with gambling disorder using gold standard clinical interviews (SCI-GD). All self-report questionnaires were taken during their first visit before patients entered treatment at the clinic, and therefore, no measures are affected by treatment. A limitation of the study is that we only had 204 participants. A greater number would have given a better-powered study. Still, our results are in many ways supported by previous literature, therefore, we believe that we had a significant number in our patients’ group that was included in the analysis. Mainly, we were interested in a clinical population, but based on earlier studies, our results can be generalized to men and women with GD. Some were born in countries other than Sweden. Participants were all residing in Sweden, and the literature describes some differences between, for example, Asian countries and cultural perspectives (87, 88). Therefore, it is not possible to generalize all groups or cultures. Finally, the study is exploratory in nature with an open-ended hypothesis, and numerous statistical tests were performed. This increases the risk of potential type 1 error. Our findings, therefore, need to be confirmed in further studies.

In conclusion, we found in this study that men started to gamble about 10 years earlier than women. We also found that more women than men were single parents. The women were also significantly more depressed and anxious than men, and more often categorized as “emotional vulnerable gamblers” in the Blaszczynski gambling pathway model (41). This might indicate that depression and anxiety are pathways to gambling disorder and a risk factor for GD for women but not for men. Furthermore, men had significantly more alcohol and other drug problems than women and fit into the pathway category “antisocial/impulsivist gamblers.” This suggests that a problematic intake of alcohol and other drugs is a risk factor for GD specifically in men.

Gender-specific associations in a treatment-seeking population are hard to characterize due to the limited number of studies in the field (1). Our findings are important not only considering the increased GD rates in women but also in relation to the specific understanding needed for both men and women seeking treatment for GD. In Sweden, men and women with GD are often treated in clinics specialized in gambling, but they can also be treated in support groups or within social healthcare. We advise caregivers to consider specific treatment plans for a problem and pathological gambling for men and women, with considerations of psychiatric comorbidity such as depression, anxiety, alcohol, and other use of drugs and sociodemographic factors such as single parenthood.

## Data availability statement

The raw data supporting the conclusions of this article will be made available by the authors, without undue reservation.

## Ethics statement

The studies involving human participants were reviewed and approved by the Regional Ethical Review Authority in Gothenburg 764-18. The patients/participants provided their written informed consent to participate in this study.

## Author contributions

LM had the main responsibility of the writing of the manuscript, contributed to the specific knowledge in gender differences and main idea for the manuscript. MM has made all the statistical analyses and responsible for the section “Results.” EA has been responsible for all of the 208 patients, from the informed consent form to collecting and keeps track of the data. AS was principal investigator for this research and supervised the writing of the whole manuscript throughout the research process. All authors contributed to the article and approved the submitted version.
